# Validity of a novel screen for cognitive impairment and neuropsychiatric symptoms in cardiac rehabilitation

**DOI:** 10.1186/s12877-019-1177-0

**Published:** 2019-06-11

**Authors:** Dana Mohammad, Nathan Herrmann, Mahwesh Saleem, Richard H. Swartz, Paul I. Oh, Janelle Bradley, Parco Chan, Courtney Ellis, Krista L. Lanctôt

**Affiliations:** 1 Hurvitz Brain Sciences Program, Sunnybrook Research Institute, Toronto, Ontario Canada; 2 Department of Pharmacology and Toxicology, University of Toronto, Toronto, Ontario Canada; 3 Department of Psychiatry, University of Toronto, Toronto, Ontario Canada; 4 University Health Network at Toronto Rehabilitation Institute, Toronto, Ontario Canada; 5 Department of Kinesiology, University of Waterloo, Waterloo, Ontario Canada; 6 Sunnybrook Hospital, FG-08, 2075 Bayview Avenue, Toronto, ON M4N 3M5 Canada

**Keywords:** Cardiac rehabilitation, Anxiety, Depression, Apathy, Cognition, Screen

## Abstract

**Background:**

Currently, there is no composite screening tool that can efficiently and effectively assess prevalent yet under-recognized cognitive and neuropsychiatric comorbidities in patients with cardiovascular disease. We aimed to determine the validity and feasibility of a novel screen assessing cognitive impairment, anxiety, apathy and depression (CAAD screen) in those attending cardiac rehabilitation (CR).

**Methods:**

All patients diagnosed with cardiovascular disease or cardiovascular risk factors entering CR were screened as part of clinical care. A subset of those patients agreed to complete validation assessments (*n* = 127). Screen results were compared to widely accepted standards for cognition, anxiety, apathy, and depression using a modified receiver operating characteristic (ROC) and area under the curve analysis.

**Results:**

The screen was completed by 97% of participants in 10 min or less with an average completion time of approximately 5 min. Screening scores adjusted for age, sex and years of education had acceptable or excellent validity compared to widely accepted standard diagnoses: CAAD-Cog (AUC = 0.80); CAAD-Anx (AUC = 0.81); CAAD-Apathy (AUC = 0.79) and CAAD-Dep (AUC = 0.85).

**Conclusions:**

The CAAD screen may be a valid and feasible tool for detecting cognitive impairment, anxiety, apathy and depression in CR settings.

## Background

Cardiovascular diseases (CVD) account for an estimated 30% of deaths worldwide, with 80% of those due to myocardial infarction and stroke [1, 2]. Management of modifiable cardiovascular risk factors (CVRFs) such as smoking, hypertension, hyperlipidemia and type II diabetes [3, 4] and physical activity are considered an essential component in preventing a subsequent CVD event [5]. As such, referral to an exercise-based cardiac rehabilitation (CR) program is strongly recommended following a CVD event [6, 7] to reduce mortality, hospitalization and improve quality of life [5, 8, 9].

Neuropsychiatric symptoms are under-recognized but particularly important risk factors of CVD. Specifically, the presence of cognitive impairment, anxiety, apathy and depression are associated with poorer quality of life and increased mortality rates [10–12]. In addition, the presence of neuropsychiatric comorbidities may be a significant barrier to the known benefits of CR [13]. Cognitive impairment can affect up to 35% of patients with CVD [14]; executive function has been shown to be the most commonly impaired domain in this population [15]. Depression is seen in up to 40% of individuals with CVD [16, 17] while the prevalence of both anxiety and apathy in CVD is approximately 25% [15, 18]. Furthermore, in approximately 50% of CVD patients, anxiety is comorbid with depression, resulting in poorer outcomes such as increased rehospitalisation and mortality rates, compared to those with CVD alone [16, 19–22].

Best practice guidelines for cardiac care recommend routine screening for cognitive impairment, depression, anxiety and lack of motivation in those undergoing CR [23, 24]. However, previous studies have shown that neuropsychiatric symptoms are frequently missed and not treated [13]. A robust comprehensive screen can improve the identification of at-risk patients and improve utilization of available psychosocial interventions such as counseling, group therapy and stress reduction classes and/or pharmacotherapy if indicated [13]. Current guidelines recommend tools such as the Center for Epidemiological Studies Depression Scale (CES-D) to screen for depressive symptoms, but offer no recommendations for cognition, anxiety and apathy [23]. While multiple tools are available, the combined time to implement individual screens for each comorbidity is prohibitive for routine use. For example, the full Montreal Cognitive Assessment (MoCA) takes approximately 10 min to administer. Given that many CR centres intake thousands of new patients each year, a comprehensive single tool that assesses common neuropsychiatric comorbidities in CVD would be less time and resource consuming in a busy clinical setting such as CR, and would be more favourable for patient acceptability to undergo testing. In support of this, previous studies have shown that a composite screen was useful in identifying neuropsychiatric comorbidities and was predictive of long-term patient outcomes including impaired functioning and poorer community integration in patients post-stroke as well as post-operative delirium and mortality in patients post-transcatheter aortic valve implantation [25–27]. Our goal was to develop a screening tool that would distinguish between CVD patients requiring referral (screen positive), those not requiring referral (screen negative) and those who need monitoring (intermediate), which could be administered in 10 min or less with minimal training.

## Methods

This study was approved by the institutional research ethics boards at Sunnybrook Health Sciences Centre and the Toronto Rehabilitation Institute (TRI) at University Health Network. Since screening for cognitive and psychiatric comorbidities is recommended by national best practice guidelines [23], a waiver of consent to be screened was approved for patients entering CR to track screening rates and times. Consenting volunteers subsequently underwent a battery of assessments within two weeks of screening.

The CAAD Screen (**C**ognitive impairment, **A**nxiety, **A**pathy, **D**epression) (Table 1) was created using existing validated screens. We selected the 5-point delayed recall from the MoCA as a brief measure of verbal memory and the Trails B test of the MoCA to assess executive function; both domains are commonly impaired in those with CVD [28–30]. The MoCA has been recommended for the assessment of vascular cognitive impairment [31, 32] and abbreviated versions of the MoCA have been shown to have comparable classification accuracy to the standard version [33, 34]. Three items adapted from the six informant items of the General Practitioner Assessment of Cognition (GPCOG) were included to capture subjective memory complaints, a necessary symptom for the diagnosis of mild cognitive impairment [35].

**Table 1:** CAAD Screen components

Items from Mini-MoCA	
1. 5 word-delayed recall	
2. Mini-trails B, 9 lines to be drawn alternating from number to letter and letter to number.	
Score: Word Recall: 1 point given for each correctly recalled word/ Trails: 1 if the trails were completed correctly, 0 if any mistakes were made.	
Three-item GPCOG	
1. In the past year have you had any difficulties with your memory? a. Has anyone commented on this?	
2. Are you less able to manage money and financial matters than you were 5 years ago?	
3. When speaking, do you have more difficulty in finding the right word, or do you tend to use the wrong words more often than you did 5 years ago?	
Score: 0 for yes to *any* of the questions, 1 for no to all questions.	
PHQ-2	
Over the past two weeks, how often have you been bothered by the following problems?	
1. Feeling down, depressed, or hopeless.	
2. Little interest or pleasure in doing things.	
Score: Not at all = 0, Several Days = 1, More than half the days = 2, Nearly everyday = 3. Range: 0–6	
GAD-2	
Over the past two weeks, how often have you been bothered by the following problems?	
1. Feeling nervous, anxious, or on edge.	
2. Not being able to stop or control worrying.	
Score: Not at all = 0, Several Days = 1, More than half the days = 2, Nearly everyday = 3. Range: 0–6	
Apathy	
Circle the response that best describes your thoughts, feelings and actions during the past 4 weeks.	
1. I am less motivated.	
2. My interest in starting or participating in conversation or activities has decreased.	
3. My interest in the world around me has decreased.	
4. My reaction to sad or exciting events has decreased.	
5. Lack of motivation or interest has made parts of my life, such as work or socializing, significantly more difficult?	
6. Lack of motivation/interest began after I started a new medication, or right after a new medical condition?	
Score: Strongly Disagree =0, Disagree =1, Agree =2, Strongly Agree = 3. Range: 0–12 for questions 1–4, Question 5 yes = 1, Question 6 No = 1. Total range: 0–14	

Depression was assessed using the Patient Health Questionnaire-2 (PHQ-2) which consists of 2 questions, scored from 0 to 3 (0–6 total). The PHQ-2 is available within the public domain and is recommended by the Canadian best practice guidelines for the assessment of depressive symptoms. The PHQ-2 has been previously validated as a screening tool for depression in those with coronary heart disease, primary care populations and stroke [36–39] with a sensitivity of 100% and a specificity of 77% among adults aged ≥65 years [38]. Similarly, anxiety was assessed using the 2-item Generalized Anxiety Disorder Scale (GAD-2), which also consists of 2 questions. The GAD-2 reflects the core diagnostic criteria of GAD according to the Diagnostic and Statistical Manual of Mental Disorders, Fifth Edition (DSM-5) [40] and has been validated in several patient populations, including those with CVD [41]. Previous studies have demonstrated that with a cut point of two or greater, the GAD-2 has a sensitivity of 67% and specificity of 90% [40].

The 6-item apathy screen, with a maximum possible score of 14, was an abbreviated adaptation of the apathy diagnostic criteria developed by Robert et al. [42]. A two-stage sequential method was used for the apathy questionnaire; participants scoring 0 on items 1–4 were assumed to be non-apathetic and subsequent yes or no items were deemed not applicable. Items 1–4 were scored on a Likert scale ranging from 0 to 3. Items 5 was scored as 1 if it was rated ‘yes’ and item 6 was scored as 1 if it was rated ‘no’. The total score was the sum of all questions. While this abbreviated version has not been used in a CVD population, apathy has been shown to be a strong, independent risk factor for CVD [12, 15, 43] and the use of apathy screening tools is well documented in various populations [12, 44].

Between June 2016 – August 2017, the CAAD screen was administered in a quiet room to all new referrals to the CR program at TRI who were able to speak and understand English and were diagnosed with CVD or CVRFs. Trained research personnel administered the screen using a script to ensure consistency. The 5-word recall and Trails B portions of the screen were administered as interview-based assessments, while the GPCOG, PHQ-2, GAD-2 and 6-item apathy questionnaire were self-administered. Research personnel remained in the room during the screening process to answer questions or clarify any confusion.

While the screen was part of clinical care, a subgroup of screened patients provided written informed consent to participate in validation testing by completing a neuropsychological battery. Cognition was assessed using the 30 min battery recommended by the National Institute of Neurological Disorders Stroke and the Canadian Stroke Network (NINDS-CSN) for the study of vascular cognitive impairment [45]. Tests included the California Verbal Learning Test 2nd Edition (CVLT-II), The Trail Making Test Part B, the Controlled Oral Word Association Test (COWAT) of phonemic fluency and the Stroop test. For all cognitive tasks, a Z-score was determined from published age, sex and education matched norms. Z-scores of tests assessing performance in the same cognitive domain can be summed into composite Z-scores as has been previously reported [46]. The Z-scores of the immediate recall, short delay free recall and long delay free recall of the CVLT-II were combined to create a composite memory score. The Trail Making Test Part B, Stroop, and COWAT Z-scores were combined to create a composite executive function score. Impairment in either cognitive domain was defined by a Z-score ≤ 1.5 standard deviation below the mean [47].

The Structured Clinical Interview for DSM-5 (SCID) disorders was used as it is a widely accepted standard to assess depression and anxiety. Those classified as major depressive disorder (MDD) by the SCID depression module (mood assessed over the last month) were considered to have depression. Similarly, those classified as generalized anxiety disorder (GAD) according to the SCID anxiety module (mood assessed over the last 6 months) were considered to have anxiety. The Apathy Evaluation Scale self-report version (AES-S) was used as the widely accepted standard for the assessment of apathy. The AES is rated on a 4-point Likert scale from 1 (not at all characteristic) to 4 (very characteristic), with higher scores indicating higher levels of apathy [48]. Participants who scored ≥37 were classified as apathetic [49].

The primary outcome was the sensitivity and specificity of the cognitive impairment, depression, anxiety and apathy components of the screen calculated using optimized cut-offs. In order to have 5 participants per item and avoid random error [50], a minimum sample size of 100 participants was targeted for the CAAD screen validation. The secondary outcome was feasibility, defined as 85% of participants completing the screen in 10 min or less [25, 51].

Statistical analyses were performed with IBM SPSS Statistics Version 24.0. Demographics for continuous variables were reported as mean ± standard deviation or number of participants and percentage for categorical variables. Agreement between the CAAD screen components and widely accepted standard assessments were evaluated using receiver operating characteristic (ROC) curves and the area under the curve (AUC) were used to determine optimized cut-offs. An AUC value of 0.8–0.9 was considered excellent and 0.7–0.8 was considered acceptable [52, 53]. While sensitivity and specificity have commonly been evaluated with one cut-off, this approach may favour optimal sensitivity over specificity, which would decrease the accuracy of the CAAD screen to identify those without neuropsychiatric symptoms. A two cut-off approach classifies participants into high, moderate and low risk groups with one cut-off maximizing sensitivity and a second cut-off with a maximum specificity, as previously reported [25]. This approach can create categories relevant to clinical care by identifying– a homogeneous low risk group unlikely to have neuropsychiatric symptoms, those with moderate risk, with possible neuropsychiatric symptoms, who should be further assessed or monitored, and those with high risk of neuropsychiatric symptoms for whom management and treatment may need to be prioritized and/or expedited [54]. Positive predictive value (PPV; true positive/true positive + false positive) and negative predictive value (NPV; true negative/true negative + false negative) were also calculated. Logistic regressions using relevant clinical variables including age, sex and years of education were applied to the ROC curves to control for these factors when predicting outcomes.

Internal consistency for each CAAD screen component was determined using Cronbach’s alpha values, where a Cronbach’s alpha ≥0.90 is excellent, 0.80–0.89 is good, 0.70–0.79 is fair and < 0.70 is unacceptable [55]. For the apathy component, since items 5 and 6 were sub-questions only to be rated if the response to the preceding questions was positive, these items were not included in the analysis.

## Results

**Fig. 1:**
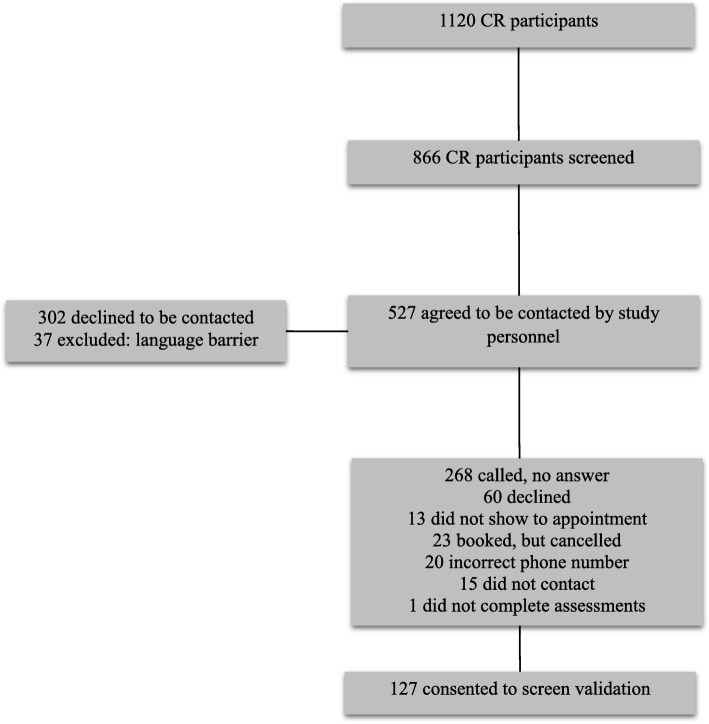
Participant selection process

The participant inclusion process is shown in Fig. 1. During the course of the study, 1120 new referrals meeting eligibility criteria were identified. Of these 254 refused screening or were missed (multiple patients seen by different physicians with limited research personnel). Screens were administered clinically to 866 patients who were subsequently approached to complete detailed testing. Of these, 527 agree to be contacted to undergo validation testing and 127completed validation testing. Participant demographics of the validation population are represented in Table 2. Included subjects were similar in terms of age (63 ± 11 versus 63 ± 13), cardiac diagnoses (MI 25% versus 25%; CABG 17% versus 13%; Stent 35% versus 34%) and vascular risk factors (hypertension 42% versus 44%; BMI 29.2 ± 6.2 versus 28.6 ± 5.9) when compared to participants who were screened only (*n* = 603). The group that completed validation testing had a significantly higher proportion of males (75.3%) compared to those that were screened only (65%) (χ^2^ = 9.1, *p* = 0.002). The screen scores for the 127 participants were as follows: the mean word recall score was 3.96 ± 1.08, 106 (84%) completed the Trails B correctly, the mean three-item GPCOG score was 1.06 ± 1.01, the mean depression score was 1.02 ± 1.38, the mean anxiety score was 0.99 ± 1.35 and the mean apathy score was 3.20 ± 3.22.

**Table 2:** Demographic Characteristics of Validation Participants (*n* = 127)

Characteristic	*n* = 127, mean ± SD or n (%)
Sociodemographics	
Age	62.4 ± 10.4
Sex (male)	94 (74)
Ethnicity (Caucasian)	93 (73.2)
Marital status, married	85 (66.9)
Total years of education	16 ± 3
Cardiac History	
Myocardial Infarction	41 (32.3)
Coronary artery bypass graft	27 (21.3)
Percutaneous transluminal coronary angioplasty	50 (39.4)
Atrial Fibrillation	10 (7.9)
Cardiomyopathy	11 (8.7)
Valvular Heart Disease	10 (7.9)
Cerebrovascular Accident	6 (4.7)
Vascular Risk Factors	
Smoking History (% quit smoking)	58 (45.7)
Hypertension	46 (36.2)
Hypercholesterolemia	26 (20.5)
Diabetes	28 (22.0)
Obese (Body Mass Index > 30 kg/m^2)^	41 (32.8)

### Validity analyses

#### Cognitive impairment

**Fig. 2:**
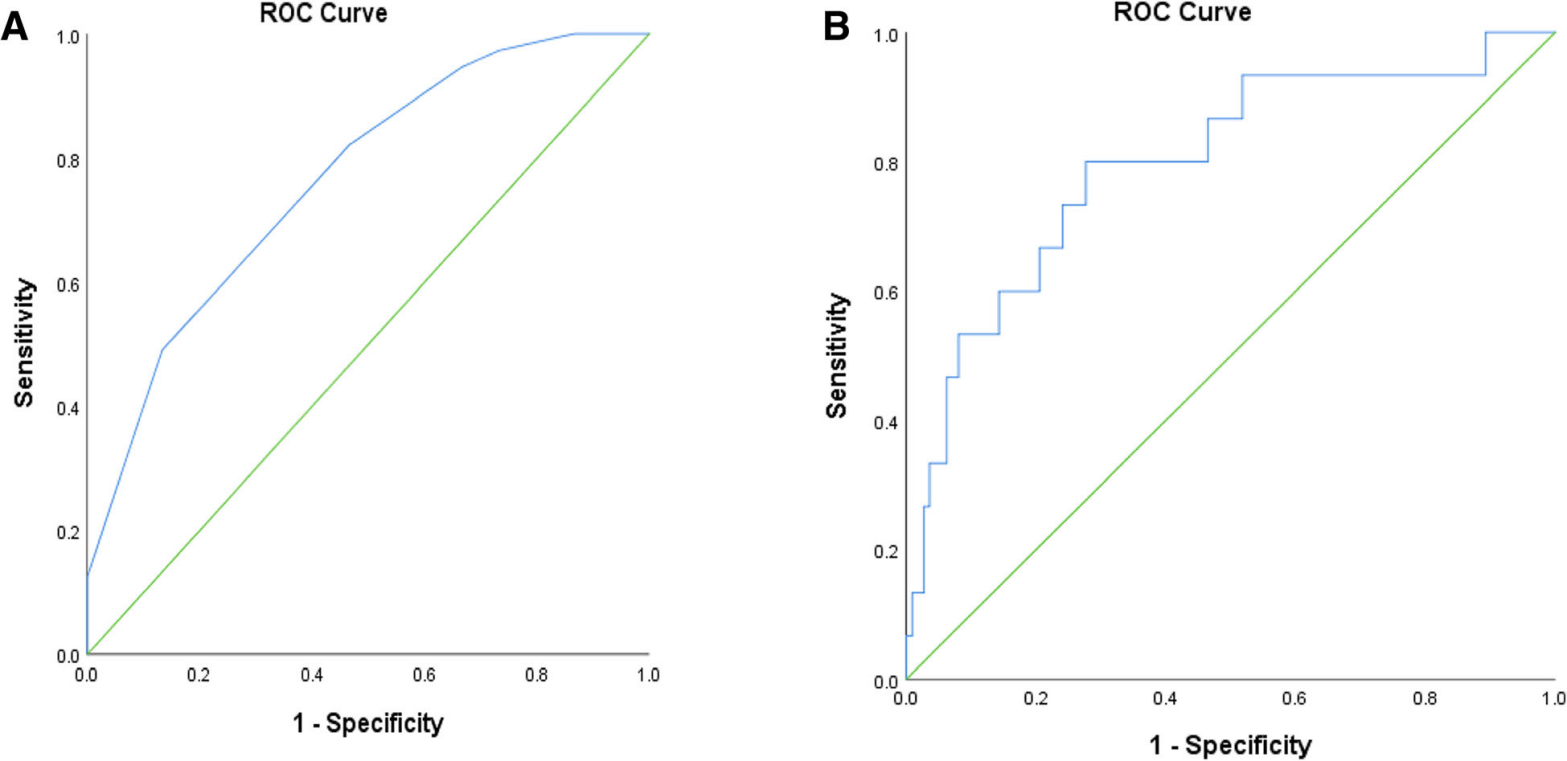
Unadjusted (**a**) and adjusted (**b**) ROC curves for the cognitive impairment screen (AUC = 0.77 and 0.80, respectively). **b** was adjusted for age, sex and total years of education

A total of 127 participants completed the NINDS-CSN cognitive battery. Of these, 15 participants (11.8%) were determined to have impaired memory and/or executive function. The ROC curves for validation of the cognitive component of the CAAD screen are shown in Fig. 2a and b. Using the two-cut point approach, the unadjusted AUC for the CAAD-Cog was 0.77 (Table 3); the sensitivity was optimized with CAAD-Cog = 6–7 (sensitivity = 95%, specificity 67%, NPV = 97%) and specificity was optimized with CAAD-Cog < 4 (specificity = 87%, sensitivity 49%, PPV = 46%). According to the CAAD-Cog, 9% were classified as high risk for cognitive impairment and 45% were classified as low risk for cognitive impairment. A subset of participants scored 4–5, and were classified as moderate risk for cognitive impairment. Of those in the moderate risk group, 14% were cognitively impaired based on the NINDS-CSN battery. When a logistic regression was applied to the ROC curve analysis controlling for age, sex and years of education, AUC was 0.80 (Table 4) and sensitivity and specificity remained high (sensitivity = 93%, specificity = 97%). The PPV improved from 46 to 57%.

**Table 3:** Unadjusted diagnostic characteristics of the CAAD screen components

Diagnostic characteristics unadjusted	Cognition	Anxiety	Apathy	Depression
AUC	0.77	0.79	0.79	0.84
Cut-off low risk	6–7	0	0–1	0
Sensitivity	95%	86%	94%	89%
Specificity	67%	42%	41%	39%
Cut-off high risk	< 4	5–6	10–14	5–6
Sensitivity	49%	10%	13%	11%
Specificity	87%	99%	95%	100%
Low risk: n (%)	57 (45%)	65 (51%)	47 (38%)	68 (54%)
SD ≤ 1.5	2	3	1	2
SD > 1.5	55	62	46	66
NPV	97%	95%	98%	97%
Moderate risk: n (%)	59 (47%)	59 (47%)	70 (56%)	55 (43%)
SD ≤ 1.5	8	16	13	12
SD > 1.5	51	43	57	43
PPV	N/A	N/A	N/A	N/A
High risk: n (%)	11 (9%)	3 (2%)	8 (6%)	4 (3%)
SD ≤ 1.5	5	2	2	4
SD > 1.5	6	1	6	0
PPV	46%	67%	25%	100%

**Table 4:** Adjusted diagnostic characteristics of the CAAD screen components

Diagnostic characteristics adjusted	Cognition	Anxiety	Apathy	Depression
AUC	0.80	0.81	0.79	0.85
Cut-off low risk	POI < 0.02	POI < 0.068	POI < 0.090	POI < 0.035
Sensitivity	93%	95%	94%	94%
Specificity	55%	39%	62%	41%
Cut-off high risk	POI > 0.30	POI > 0.76	POI > 0.43	POI > 0.57
Sensitivity	27%	5%	13%	50%
Specificity	97%	99%	97%	99%
Low risk: n (%)	55 (43%)	42 (33%)	69 (55%)	47 (37%)
SD ≤ 1.5	1	1	1	1
SD > 1.5	54	41	68	46
NPV	98%	98%	99%	98%
Moderate risk: n (%)	65 (52%)	83 (65%)	51 (41%)	70 (55%)
SD ≤ 1.5	10	19	13	8
SD > 1.5	55	64	38	62
PPV	N/A	N/A	N/A	N/A
High risk: n (%)	7 (6%)	2 (2%)	5 (4%)	10 (8%)
SD ≤ 1.5	4	1	2	9
SD > 1.5	3	1	3	1
PPV	57%	50%	40%	90%

#### Anxiety

**Fig. 3:**
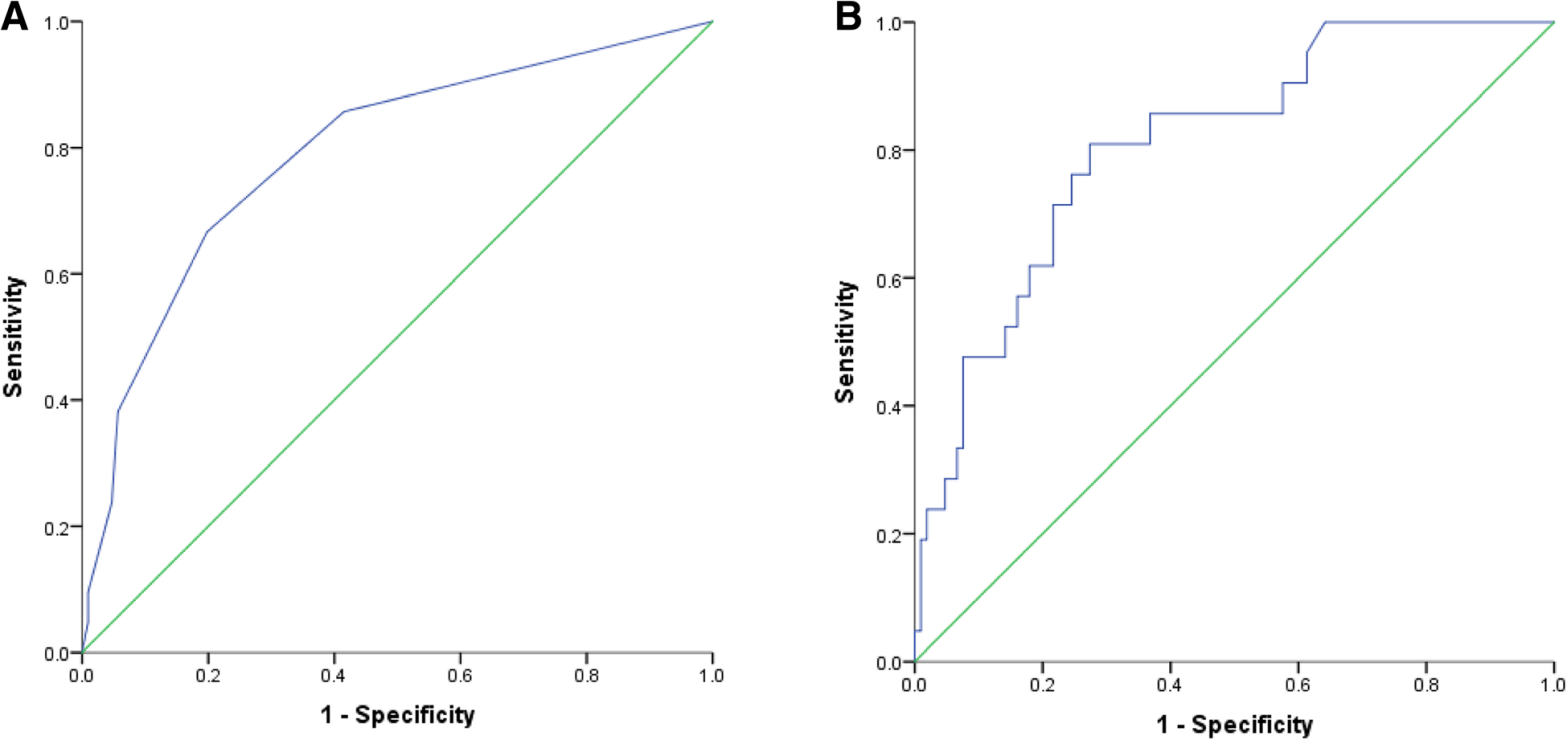
Unadjusted (**a**) and adjusted (**b**) ROC Curves for the anxiety screen (AUC = 0.79 and 0.81, respectively). **b** was adjusted for age, sex and total years of education

A total of 127 participants completed the SCID anxiety module. Of these 16.5% were classified as having GAD. The ROC curves for the validation of the anxiety portion of the screen are shown in Fig. 3a and b. The unadjusted AUC was acceptable (0.79); sensitivity was optimized with a CAAD-Anx score of 0 (sensitivity 86%, specificity 42%, NPV = 95%) and specificity was optimized with a score ≥ 5 (specificity = 99%, sensitivity 10%, PPV = 67%) (Table 3). The screen identified 2% of the participants as high risk and 51% as low risk for anxiety. A total of 47% were classified as moderate risk. According to the SCID-GAD module, 17% of participants had GAD, and 27% of those in the moderate risk group had GAD. The adjusted AUC was 0.81 and both sensitivity and specificity remained high (95 and 99% respectively). The PPV was highest at 67% using the unadjusted two cut-off method, while the NPV was highest at 98% using the adjusted two cut-off method (Table 4).

#### Apathy

**Fig. 4:**
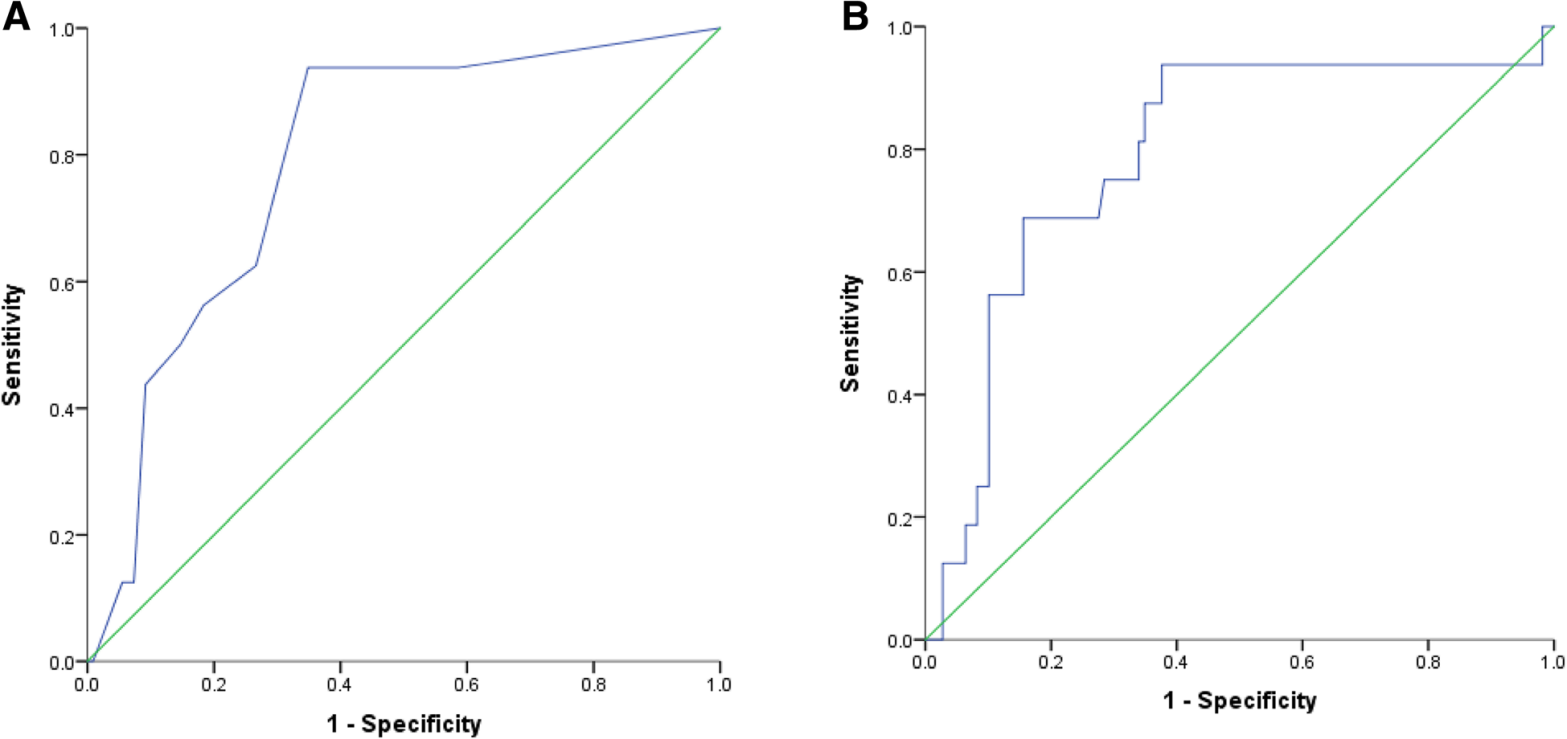
Unadjusted (**a**) and adjusted (**b**) ROC Curves for the apathy screen (AUC = 0.79 and 0.79, respectively). **b** was adjusted for age, sex and total years of education

A total of 125 participants completed the AES-S scale. The ROC curves for the validation of the apathy portion of the screen are shown in Fig. 4a and b. The unadjusted AUC was acceptable at 0.79 (Table 3); the sensitivity was optimized at CAAD-Apathy = 0–1 (sensitivity = 94%, specificity 41% NPV = 98%) and specificity was optimized at CAAD-Apathy ≥10 (specificity = 95%, sensitivity 13%, PPV = 25%). According to the screen, 6% of participants were at high risk for apathy while 38% were at low risk for apathy. The remaining 56% scored 2–9 and were classified as moderate risk. According to the AES, 13% had clinically significant apathy and 19% of those in the moderate risk group were apathetic. While the AUC did not change after adjustment for age, sex and years of education, the NPV and PPV were both highest when the adjusted two cut-off method was applied (99 and 40%, respectively) (Table 4).

#### Depression

**Fig. 5:**
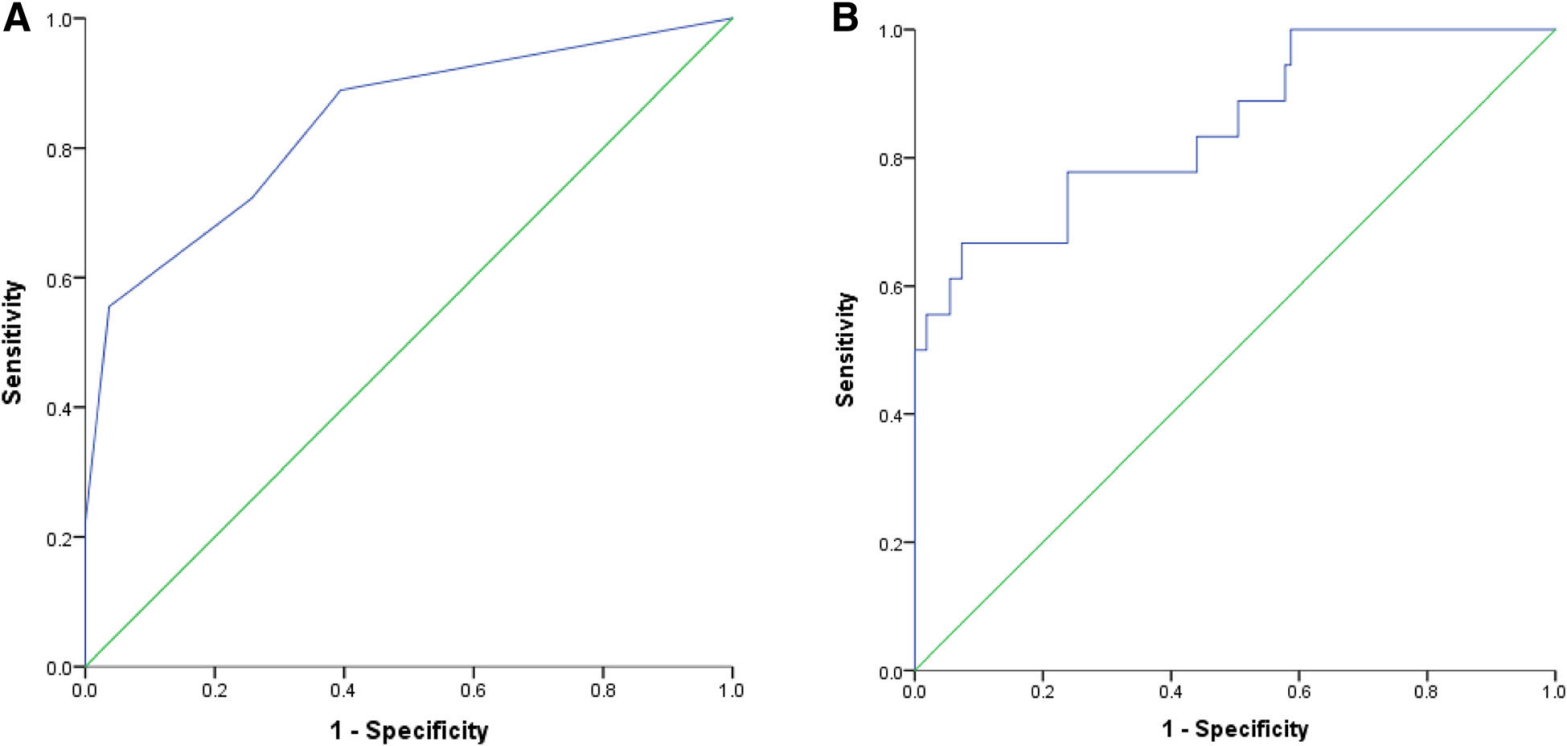
Unadjusted (**a**) and adjusted (**b**) ROC Curves for the depression screen (AUC = 0.84 and 0.85, respectively). **b** was adjusted for age, sex and total years of education

Of the 127 participants who completed the SCID depression module, 14.2% had major depression. The ROC curves for the depression validation are shown in Fig. 5a and b. The unadjusted AUC for the CAAD-Dep was 0.84; the sensitivity was optimized at CAAD-Dep = 0 (sensitivity = 89%, specificity 39%, NPV = 97%) and specificity was optimized at CAAD-Dep ≥ 5 (specificity = 100%, sensitivity 11%, PPV = 100%) (Table 3). The depression screen indicated 3% of participants as having high risk for depression while 54% were classified as having low risk for depression. The remaining 43% scored 1–4 on the screen and were classified as having moderate risk of depression. According to the SCID depression module, 14% were confirmed as depressed and of those in the moderate risk group, 22% were classified as depressed. The AUC adjusted for age, sex and years of education was 0.85 and both the sensitivity and specificity remained high (sensitivity = 94% and specificity = 99%) (Table 4).

### Feasibility and internal consistency

Of 102 participants where time was recorded, 97.1% completed the screen in under 10 min with a mean (SD) of 4.9 (1.7) minutes. The internal consistency was good for depression, anxiety and apathy (items 1–4) but unacceptable for the three-item GPCOG (Table 5).

**Table 5:** Internal Consistency for each screen component

	Depression	Anxiety	Apathy (Q 1–4)	GPCOG
Cronbach’s α	0.83	0.86	0.84	0.69

## Discussion

This study assessed the validity and feasibility of the CAAD screen, a brief tool to assess risk for cognitive impairment, depression, anxiety and apathy in a CR setting. Current practices for comorbidity screening in a CR setting are not standardized. Specifically at TRI, participants are screened for depressive symptoms using the CES-D [13]. While the CES-D is a well validated and widely used screening tool, it can take up to 10 min to administer [56]. As such, additional time would be needed to obtain data for other important neuropsychiatric comorbidities, which would be burdensome in a busy clinical setting such as CR. Hence, a single tool to measure all four neuropsychiatric comorbidities in a timely manner would be clinically useful.

The CAAD screen was validated using the multiple ROC curve cut-off approach published previously [25]. Study findings show that all components of the screen demonstrated acceptable or excellent diagnostic characteristics. CAAD-Cog displayed strong sensitivity and specificity for detecting cognitive impairment using the two-cut point approach and adjusting for clinical factors (Fig. 2b). Of the large proportion (51%) of participants categorized as moderate risk, 15% of those were impaired according to the NINDS-CSN battery. While up to 35% of patients with CVD may experience cognitive impairment [14], only 12% of participants in this study scored ≤1.5 SD below the mean on tests of memory and/or executive function. We used a cut-off of ≤1.5 SD as it is a more stringent and frequently used cut-off than ≤1 SD [47]. However, if we had chosen a cut-off of ≤1 SD indicative of subtle deficits as has been done before in this population [46], 24% of our participants would have been classified as cognitively impaired, which more closely reflects the prevalence in the literature.

The NPV of the cognition section was excellent, while the PPV was only acceptable. This may suggest that this portion of the screen is likely to incorrectly classify those without cognitive impairment and is more suitable for detecting those who are at a low risk. Studies have demonstrated that there is often a high rate of false positives associated with brief cognitive screens and measures must be taken to find an optimal trade-off between speed and accuracy [57–59]. To reduce time and costs associated with extensive neurological follow-up, studies have recommended the administration of a second cognitive screen and that a firm diagnosis be made only if cognitive impairment was evident in multiple domains assessed by validated neuropsychological measures [57, 60].

The CAAD-Anx displayed excellent sensitivity and specificity for detecting GAD using the two cut-point approach. The screen correctly identified two-thirds of those in the high risk group, leaving a large proportion of participants classified as moderate risk similarly to other studies that have screened for anxiety and psychological distress in a CR population [61, 62]. Completing follow-up assessments for all patients in the moderate risk group would add a significant time burden for physicians and patients who ultimately do not have GAD. However, 27% of patients with GAD classified into this group. For those classified as moderate risk, CR staff may consider reassessment after 4 weeks [62] or completing a shorter anxiety screen, such as the GAD-7, before proceeding with a full length follow up assessment.

In comparison to the other portions of the screen, the CAAD-Apathy ROC curve did not show an improved AUC value when adjusted for age, sex and total years of education; however, adjusting for clinical variables reduced the number of participants who scored intermediate-risk from 56 to 41%, while remaining acceptable with excellent sensitivity and specificity. Adjustment for clinical factors also increased the PPV from 25 to 40% suggesting more accurate classification of patients. Previous studies indicated that ethnic differences are associated with apathy and therefore this factor may contribute to improvements in the AUC [63, 64].

The PHQ-2 had the best AUC values in comparison to other screen sections. Both the NPV and PPV were excellent, with 100% of those classified into the high risk group being diagnosed as depressed by the SCID-5. Furthermore, of the 18 participants classified as depressed according to the SCID depression module, only two were categorized into the low risk group. These robust diagnostic characteristics of the PHQ-2 support use of this tool depression in this population. Furthermore, due to the high prevalence and negative health outcomes associated with depressive symptoms in those undergoing CR [13], routine screening will allow referral to healthcare providers to confirm diagnosis and initiate treatment.

While there may be overlap between the screen components of the depression and apathy components of the screen, depression and apathy are now recognized as independent clinical syndromes [65]. They may also influence participation in CR. As previously shown, depression decreases completion and adherence in CR [13]. In addition, we expect that the lack of motivation and interest associated with apathy may also lead to less participation in CR. Hence, the accurate identification of which neuropsychiatric syndrome is impeding participation is critical to inform strategies to improve attendance and adherence to CR.

All components of the screen classified a large proportion of participants into the moderate risk group consistent with previous reports of subtle cognitive deficits and neuropsychiatric symptoms in this population [61, 66–68]. While following up with this group would ensure that all participants at risk receive follow up, it may cause those who are incorrectly classified to endure short-term psychological consequences or require physicians to conduct time-consuming assessments. As such, when screening CR participants for these comorbidities, those who score as being at moderate risk may be appropriate for close monitoring and further clinical assessment if necessary.

The CAAD screen was implemented as part of routine clinical care and was completed by all participants suggesting adequate patient acceptability. An overall mean time of completion of 4.9 min indicates that the CAAD screen is efficient in capturing clinically relevant information for all four CVD-related neuropsychiatric comorbidities in a large-volume clinic. Out of 127 participants, the CAAD screen identified 20 for follow-up, which is a feasible number at our site, leaving the majority in the monitoring and no symptoms group.

Findings from this study are consistent with other studies using a combined screen to detect presence of important comorbidities in other populations. For example, the unadjusted AUC for the depression screen (PHQ-2) in the present study was comparable to the AUC for the PHQ-2 in patients with stroke screened for depressive symptoms (0.84 vs. 0.90) [25]. The optimized sensitivity (89% vs. 92%) and specificity (100% vs. 99%) using a two cut-off approach were also similar between the two studies.

Limitations of the study include a single centre study design and the inability to compare adapted screen components to norms. Participants had an average age of 62 years, 16 years of education and were predominantly Caucasian and male. While representative of the CR population [69, 70], the study sample may not represent all patients with CVD. However, results were adjusted for demographic characteristics such as age, sex and years of education and the full versions of the screen components have been validated in diverse populations [71–73], which may improve generalizability. In addition, CR participants may be more motivated to exercise and participate in research studies. The possibility that a selection bias in favour of higher functioning and more motivated patients might have been introduced at the recruitment level cannot be ruled out and may explain the lower than expected incidence of neuropsychiatric symptoms in this study. It is important to implement the CAAD screen in multiple clinical centres to determine its generalizability and validity. In addition, although the internal consistency of the depression, anxiety and apathy (questions 1–4) portions of the screen were good, the GPCOG questions included in the CAAD screen used to assess mild cognitive impairment, may not be the most applicable to CR participants, who generally present with subtle early cognitive changes. Other questions on the full length GPCOG, such as trouble recalling conversations a few days later or difficulties in managing medication independently, may be more suitable and future studies should consider these questions as a measure of subjective cognitive function [74]. Future studies could also explore the assessment of biometrical quality of the individual screen items as an alternative approach for the selection of questions in the screening tool.

## Conclusions

The CAAD screen had acceptable validity and feasibility for assessing prevalent yet under-recognized comorbidities in those with CVD or CVRFs attending CR. Use of the two cut-off approach resulted in optimized sensitivity and specificity for all portions of the screen and had clinical applicability by identifying those who needed further follow-up, and those who did not. Screening for these important and prevalent neuropsychiatric comorbidities in CVD patients and those with CVRFs can identify patients at risk for non-completion of CR and those who may need additional management.

## Data Availability

The datasets generated and/or analysed during the current study are not publicly available since patient consent was not obtained for publication or sharing of patient-level data.

## Abbreviations

AES: Apathy Evaluation Scale
AUC: Area under the curve
CAAD: Cognitive impairment, Anxiety, Apathy, Depression
CES-D: Center of epidemiological studies depression scale
CR: Cardiac rehabilitation
CVD: Cardiovascular disease
CVRFs: Cardiovascular risk factors
DSM-5: Diagnostic and Statistical Manual of Mental Disorders, Fifth Edition
GAD-2: Generalized Anxiety Disorder-2
GPCOG: General Practitioner Assessment of Cognition
MoCA: Montreal Cognitive Assessment
NINDS-CSN: National Institute of Neurological Disorders Stroke and the Canadian Stroke Network
NPV: Negative Predictive Value
PHQ-2: Patient Health Questionnaire-2
POI: Probability of impairment
PPV: Positive Predictive Value
ROC: Receiver operating characteristic
SCID-5: Structured Clinical Interview for DSM-5
